# Obesity Impact Evaluated from Fat Percentage in Bone Mineral Density of Male Adolescents

**DOI:** 10.1371/journal.pone.0163470

**Published:** 2016-09-29

**Authors:** Wagner Luis Ripka, Jhomyr Dias Modesto, Leandra Ulbricht, Pedro Miguel Gewehr

**Affiliations:** 1 Graduate Program in Electrical Engineering and Computer Science, Federal University of Technology-Paraná, Curitiba, Brazil; 2 Graduate Program in Biomedical Engineering, Federal University of Technology-Paraná, Curitiba, Brazil; University of Arkansas for Medical Sciences College of Pharmacy, UNITED STATES

## Abstract

**Objective:**

To analyze bone mineral density (BMD) values in adolescents and to assess obesity impact, measured through body fat #x2013;on this variable through the assessment by DEXA.

**Methodology:**

A total of 318 males adolescents (12–17 years) were evaluated considering weight, height, body mass index (BMI), bone mineral density (BMD), fat and lean mass. BMD was assessed for the arms, legs, hips, and lumbar regions, as well as for total amount. Stratification of the nutritional status was determined by body fat (%BF) percentage; comparison of groups was scrutinized by analysis of variance; and the association of variables was performed using Pearson's test.

**Results:**

There was a progressive increase in weight, height, and BMD for all evaluated age groups following the advance of chronological age. A negative correlation was found between the %BF with BMD in all evaluated segments. Significant differences were found between the eutrophic group compared to the overweight group and the obesity group in the evaluated segments (P <0.01) noting a reduction of up to 12.92% for the lumbar region between eutrophic and obese.

**Conclusion:**

The results suggest that increase %BF is associated with lower BMD among male adolescents.

## Introduction

Adolescence represents a critical period for bone mineralization in both genders [1] and approximately 60% risk of developing osteoporosis in adulthood is related to bone mass acquisition in this stage of life [2,3]. However, there are several factors associated with changes in this tissue, including age [1], nutritional and, hormonal status [4,5], physical exercise [6], genetics and dietary habits [7,8].

In regard to nutritional status, the excess of fat during adolescence, in addition to effects on bone tissue [4,9], is largely related to the onset of clinical manifestations of coronary heart disease [10], respiratory problems [11] and type 2 diabetes [12]. These risk factors associated with obesity and its alarming growth in recent decades has put the control of this disorder as a new health policy challenge worldwide, even in developing countries [13].

Among the ways to evaluate bone tissue and fat tissues there is the technology of dual energy X-ray absorptiometry (DEXA). This method, developed in the 80s, has its operation based on the measurement of photons attenuation generated by X-ray sources, in both low and high density tissues, allowing the assessment of bone tissue characteristics such as mineral density (BMD) (g/cm^2^) and mineral content (g). As for the body mass, the equipment allows fat mass section, lean mass section and body fat percentage, and it is considered one of the main evaluation methods [14,15].

Previous studies [4,16–18] which evaluated the relationship between BMD and nutritional status used body mass index (BMI) as a ranking factor, but it is believed that this method tends to generate a different interpretation of that proposed by the analysis of body fat specifically.

Thus, the objective of this study was to analyze BMD values in male adolescents (12–17) and to verify the impact of obesity as measured by body fat on the BMD variable through measurements by DEXA.

## Materials and Methods

### Study population

Data for this study were obtained for approximately two years (2015–2016) among caucasian adolescents enrolled in the school system, aged 12 to 17 whose parents gave them permission to participate in the research by signing an informed consent form. This study was approved by the Ethics Committee through the Brazil Platform under number 11583113.7.0000.5547.

A sampling error of 4% under confidence level of 95% for a population of 82,414 individuals was specified for the sample size [19]. All adolescents whose: a) parents did not allow participation; b) made use of medicines containing calcium; c) went through radiography in the seven days before the evaluation, were excluded from the research.

### Anthropometric assessment

Body mass was collected with the use of a mechanical scale and height with the use of a stadiometer coupled to the scale (Filizola, São Paulo, SP, Brazil).

The DEXA evaluation was performed using a Hologic Discovery A fan-beam scan type (Hologic, Inc., Bedford, MA, USA). Individuals were placed in supine position on the scanner table. It was not allowed the presence of metal artifacts such as earrings, chains or rings, as well as clothing possessing any kind of metal. The total fat percentage (%BF) was obtained automatically by the machine software. For BMD values, five evaluation points were considered: arms, legs, pelvis, lumbar and whole body.

The nutritional classification for %BF was made based on the criteria established by Lohman et al. [20]. Boys with values up to 20% were considered eutrophic; >20% and ≤25% overweight and >25% obese. With regard to the BMI classification, it was made from the index calculation (kg/m^2^) using the recommendation of the World Health Organization (WHO) which classifies as eutrophic adolescents those between the 5th and the 85th percentiles, as overweight those classified between the 85th and the 95th percentiles, and as obese those above [21].

### Statistical analysis

Statistical analysis was made by descriptive presentation of the mean ± standard deviation values. To quantify the intensity and direction of variables association the Bravis-Pearson bivariate correlation test was performed. To test for the equality of mean BMD measurements between nutritional groups (eutrophic, overweight, obese) we used a one-way analysis of variance (ANOVA). When *H*_0_ was rejected and the existence of a difference was found between sample groups, the post-Hoc Tukey test was applied for multiple comparisons. The assumption of normal distribution of variables was tested using Kolmogorov-Smirnov.

For the analysis, the statistical significance value p < 0.05 was adopted and the statistical packages –Statistical Package for Social Sciences (SPSS)–version 17.0 (SPSS Inc. Chicago, IL) were used.

## Results

Three hundred and eighteen adolescents were evaluated with an average age of 14.87±1.52. For normal distribution analysis, p > 0.05 was obtained in all variables. Descriptive values for anthropometric variables and bone mineral density are shown in Table 1. It is observed a progressive increase in weight (kg), height (m) and BMD (g/cm^2^) following chronological age advance. The characteristics were presented in mean±standard deviation form.

**Table 1:** Body composition and bone mineral density characteristics according to chronological age (Mean±Standard Deviation).

Ages/Variables	12–13	14–15	16–17	Total
	(n = 61)	(n = 135)	(n = 122)	(n = 318)
General Body Composition				
Weight (kg)	50.8±12.3	60.1±9.8	66.2±10.7	60.7±12.0
Height (m)	1.6±0.1	1.7±0.1	1.7±0.1	1.7±0.1
BMI (kg/m2)	20.4±3.6	20.9±2.7	22.2±5.5	21.3±4.2
Fat Mass (kg)	13.5±6.0	13.2±9.5	13.2±5.0	13.3±7.4
Lean Mass (kg)	36.8±9.9	50.1±36.7	52.1±20.4	48.4±28.0
Body Fat (%)	24.9±6.5	19.1±4.0	18.7±4.6	20.1±5.3
Bone Mineral Density				
Pelvis (g/cm2)	1.2±0.1	1.4±0.1	1.5±0.1	1.2±0.2
Arms (g/cm2)	1.3±0.1	1.4±0.1	1.5±0.1	1.4±0.2
Legs (g/cm2)	2.0±0.2	2.3±0.2	2.5±0.3	2.3±0.3
Lumbar (g/cm2)	1.4±0.2	1.7±0.2	1.9±0.2	1.7±0.3
Whole Body (g/cm2)	0.9±0.1	1.1±0.1	1.2±0.1	1.1±0.1

**Fig 1:**
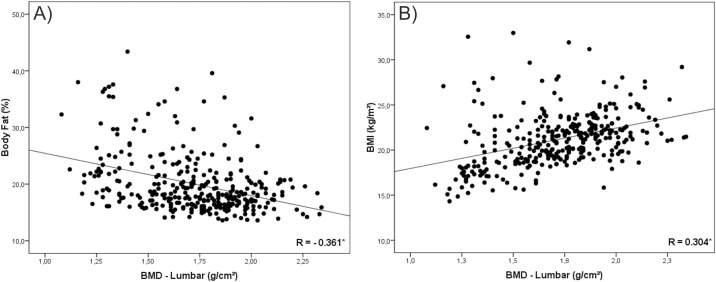
Graphical correlation analyses between Bone Mineral Density –Lumbar and Nutritional Status. (A) Stratification from %BF. (B) Stratification from BMI.

**Fig 2:**
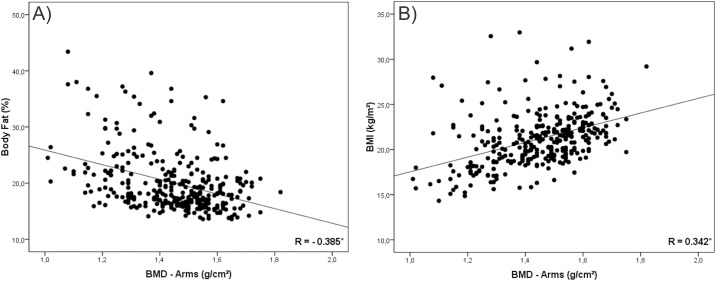
Graphical correlation analyses between Bone Mineral Density –Arms and Nutritional Status. (A) Stratification from %BF. (B) Stratification from BMI.

**Fig 3:**
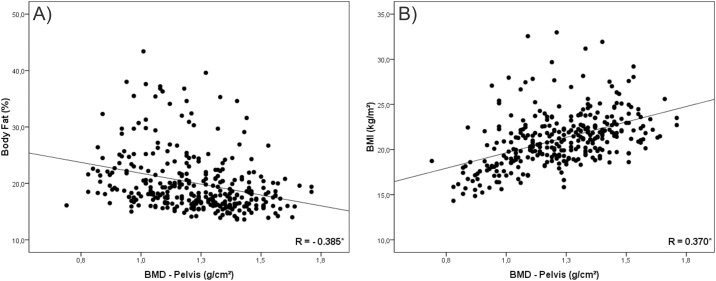
Graphical correlation analyses between Bone Mineral Density –Pelvis and Nutritional Status. (A) Stratification from %BF. (B) Stratification from BMI.

**Fig 4:**
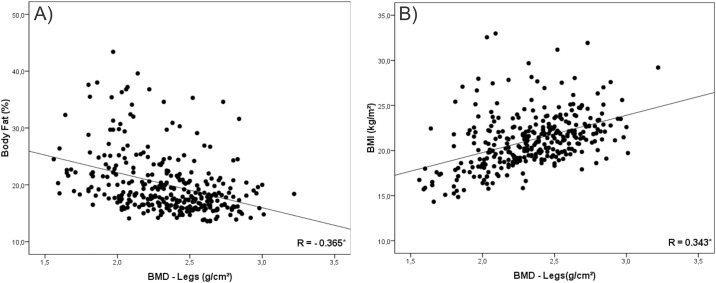
Graphical correlation analyses between Bone Mineral Density –Legs and Nutritional Status. (A) Stratification from %BF. (B) Stratification from BMI.

**Fig 5:**
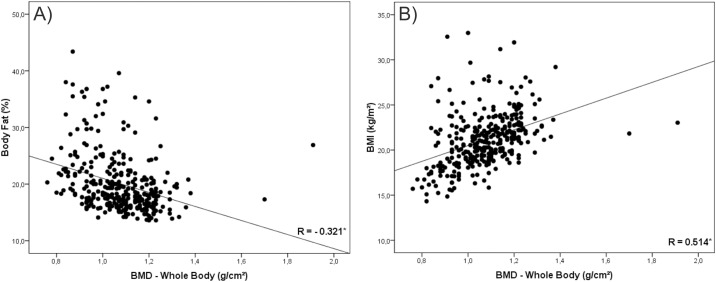
Graphical correlation analyses between Bone Mineral Density –Whole Body and Nutritional Status. (A) Stratification from %BF. (B) Stratification from BMI.

In a second instance, we tested the correlation of BMI and %BF with BMD. Our findings were that in all analysis %BF presented negative correlation with BMD (Figs 1A, 2A, 3A, 4A and 5A). As opposed to that, BMI presented positive association as shown in Figs 1B, 2B, 3B, 4B and 5B.

Using the ANOVA, with Tukey post-Hoc, to test the impact of %BF on BMD variables, we have noted a significant decrease between overweight and obesity groups in comparison with the eutrophic group in all evaluated segments. There was a reduction of up to 12.92% for the lumbar region between eutrophic and obese groups. The impact of increased %BF in BMD was also observed between overweight and obesity groups for arms and lumbar regions (Table 2 and S1 Table).

**Table 2:** Sample characterization according to nutritional status for %BF (Mean±Standard Deviation).

%BF Classification	Eutrophic	Overweight	Obesity	Difference^1^
	(n = 204)	(n = 70)	(n = 44)	
General Body Composition				
Weight	60.36±9.5*	60.89±14.81	64.90±15.63	--
Height	1.71±0.08*†	1.68±0.12	1.63±0.10	--
Fat Mass (kg)	11.44±7.12*†	15.16±6.96*	20.09±6.18	--
Lean Mass (kg)	50.26±32.60	45.92±14.64	42.73±10.79	--
Body Fat (%)	17.08±1.60*†	22.02±1.45*	30.85±4.67	--
Bone Mineral Density				
Pelvis (g/cm2)	1.26±0.20*†	1.17±0.21	1.12±0.17	-11.11%
Arms (g/cm2)	1.48±0.13*†	1.41±0.18*	1.33±0.15	-10.07%
Legs (g/cm2)	2.41±0.29*†	2.24±0.33	2.13±0.28	-11.57%
Lumbar (g/cm2)	1.78±0.24*†	1.66±0.28*	1.53±0.24	-12.92%
Whole Body (g/cm2)	1.11±0.12*†	1.04±0.14	1.00±0.18	-9.91%

^1^ Difference between eutrophic and obesity BMD

*Difference for Obesity

^†^Difference for Overweight

After the group's nutritional status was categorized from the BMI, we noted a significant increase in BMD for the overweight group in comparison to the eutrophic group for the variables pelvis and arms. When comparing the groups Overweight and Obesity, the arms and lumbar regions showed lower significant values for the obese group (Table 3 and S2 Table).

**Table 3:** Sample characterization according to nutritional status for BMI (Mean±Standard Deviation).

BMI Classification	Eutrophic	Overweight	Obesity
	(n = 232)	(n = 68)	(n = 18)
General Body Composition			
Weight	57.15±9.91*†	68.84±11.12*	64.95±14.36
Height	1.69±0.09*	1.70±0.10*	1.63±0.10
Fat Mass (kg)	11.49±6.73*†	16.81±6.33*†	23.96±5.77
Lean Mass (kg)	49.96±32.20	50.10±10.71	47.44±11.51
Body Fat (%)	18.33±3.34*†	22.72±5.30*†	32.51±5.68
Bone Mineral Density			
Pelvis (g/cm2)	1.21±0.21†	1.29±0.19	1.18±0.19
Arms (g/cm2)	1.44±0.15†	1.50±0.15*	1.39±0.20
Legs (g/cm2)	2.32±0.31	2.41±0.31	2.24±0.37
Lumbar (g/cm2)	1.71±0.26	1.77±0.24*	1.58±0.30
Whole Body (g/cm2)	1.07±0.13	1.10±0.13	1.07±0.25

*Difference to Obesity

^†^ Difference to Overweight

## Discussion

This study verified the impact of %BF in BMD in male adolescents. The results showed that group stratification according to the %BF highlights the significant loss of BMD for overweight and obesity groups as compared to the eutrophic group in all evaluated segments, with a loss of 9.91% (whole body) and 12.92% (lumbar). The findings recorded from the use of BMI as classification criteria, in turn,demonstrateda different association between nutritional status change and BMD loss.

Although the mechanism involving the relationship between adipose and skeletaltissue to be uncertain [4]. Amongst the explanations for that one can note the relation between the increase in adipogenesis and the decrease of osteoblastogenesis caused by obesity [22] in addition to diets with excess of fatty foods which interfere in calcium absorption [17,22].

Fat excess in adolescents is highlighted with concern by the World Health Organization publications, since it generates an early increase of medical expenses [21,23]. The inverse relationship between %BF and BMD pointed in this study suggests the need for a new approach to the health of adolescents. Therefore, the increase of %BF and the decrease in BMD may reflect bone metabolic disorder and, subsequently, osteoporosis. In Brazil, where ~75% of the population uses the free health care system, the creation of public policies associated with the impact of the relationship of %BF in BMD may prevent a collapse in public spending.

Recent research which compared bone measurements in adolescents according to the nutritional status from BMI diverged from the findings of this study. Mosca et al. [4] evaluated 170 boys (10–19) from São Paulo (Brazil) and showed that eutrophic individuals had lower BMD values for the lumbar, femur, and total body compared to adolescents with overweight, obesity and extreme obesity. Similar results to the previous study were found by Jeddi et al. [17] after assessing 237 Jordanian adolescents (9–18) where eutrophic, overweight and obese individuals showed mean BMD values for whole body equal to 0.88±0.11g/cm^2^, 0.93±0.11g/cm^2^, 0.93±0.12g/cm^2^, respectively. Another study evaluated 235 adolescents among which 103 were obese (4–20), and again it was found that individuals with obesity, ranked from BMI, were those with higher BMD values [24].

The divergence of results can be explained by the fact that in all cases BMI was used for nutritional classification of individuals and not the %BF. The use of this index as rating instrument has as its parameters both weight and height. Both variables have a positive correlation with the bone tissue, and BMI is limited to distinguish fat mass and muscle mass [25].

Despite this contradiction, the linear increase trend in BMD values with the advance of age and the negative association of this variable with body fat corroborates other studies conducted with adolescents [1,4,17,26]. Moreover, correlation values found between BMI and BMD (R = 0.514; P < 0.001) and between %BF and BMD (R = -0.321; P < 0.001), as well as graphical analysis, reinforce the opposition between the two classification forms [18,27].

## Conclusion

In conclusion some limitations need to be highlighted. Although all participants were white, and was not possible to keep a specific control over race of the adolescents. Considering that in the region where the study was conducted there are adolescents with ethnic background from different regions in Europe (Ukrainians, Polish, Italian, German, Portuguese and Lebanese), besides the ones with Latin American ancestry. Sexual maturation of those individuals and its impact on skeleton were not controlled. Finally, it is suggested that overweight and obesity, classified from the %BF are associated with lower BMD among male adolescents. This strengthens the need for health public policies for adolescents in order to prevent and control body fat excess.

## Supporting Information

S1 Table ANOVA, with Tukey post-Hoc, to test the impact of %BodyFat on bone mineral density variables. (XLSX)

S2 Table ANOVA, with Tukey post-Hoc, to test the impact of BMI on bone mineral density variables. (XLSX)
